# Case Report: Intranasal esketamine and accelerated intermittent theta-burst stimulation for severe treatment-resistant depression with suicidal ideation

**DOI:** 10.3389/fpsyt.2026.1837402

**Published:** 2026-07-01

**Authors:** Chun-Hung Chang, Yu-Der Hsia, Wen-Chun Liu, Chien-Ho Lin, Jianjung Ying, Hsin-Chi Tsai

**Affiliations:** 1 An Nan Hospital, China Medical University, Tainan, Taiwan; 2 College of Medicine, China Medical University, Taichung, Taiwan; 3 Department of Nursing, National Tainan Junior College of Nursing, Tainan, Taiwan; 4 Department of Psychiatry, Chi Mei Medical Center, Tainan, Taiwan; 5 Department of Aging Medicine, China Medical University, Taichung, Taiwan; 6 Department of Psychiatry, Tzu-Chi General Hospital, Hualien, Taiwan; 7 Institute of Medical Sciences, Tzu Chi University, Hualien, Taiwan

**Keywords:** accelerated transcranial magnetic stimulation, intermittent theta burst stimulation, intranasal esketamine, suicidal ideation, treatment-resistant depression

## Abstract

**Introduction:**

Treatment-resistant major depressive disorder with high-risk suicidal ideation is a psychiatric emergency that requires rapid and effective intervention. Although intranasal esketamine and intermittent theta-burst stimulation (iTBS) are time-efficient therapeutic options, clinical reports describing their combined use in an accelerated inpatient schedule are limited, particularly in acutely high-risk patients.

**Methods:**

A 29-year-old woman with recurrent major depressive disorder was hospitalized for severe depressive symptoms and high-risk suicidal ideation after inadequate responses to several antidepressant trials, augmentation therapy, psychotherapy, and a standard course of repetitive transcranial magnetic stimulation. Her baseline symptom severity scores were as follows: Patient Health Questionnaire-9 (PHQ-9), 15; Hamilton Depression Rating Scale (HAMD), 27; and Beck Depression Inventory (BDI), 41. After providing written informed consent, she received intranasal esketamine (84 mg) and an accelerated iTBS protocol targeting the left dorsolateral prefrontal cortex (Beam F3 localization), with 10 iTBS sessions delivered over 4 treatment days (total 6,000 pulses).

**Results:**

The patient’s depressive symptoms improved during the acute treatment course (PHQ-9: 15→11; HAMD: 27→20; BDI: 41→28), and she reported an improvement in suicidal ideation. Her clinical status remained stable at the 2-week follow-up (PHQ-9 = 8; HAMD = 17; and BDI = 23). An exploratory device-derived stress index obtained using the Stress Electroencephalogram Assessment (SEA) system decreased from 7 to 3, consistent with clinical improvement.

**Conclusion:**

This case suggests that the combination of intranasal esketamine and accelerated iTBS is feasible in inpatient settings for treating severe depression with high-risk suicidal ideation. Additional controlled studies are required to evaluate the efficacy, optimal sequencing, safety monitoring, and durability of this combined approach.

## Introduction

Major depressive disorder is a leading cause of disability (1). In particular, major depressive disorder accompanied by suicidal ideation represents a high-risk clinical state that often requires immediate interventions (2). However, a substantial proportion of patients do not achieve adequate symptom relief despite sequential trials of antidepressant pharmacotherapy, psychotherapy, and neuromodulation. Therefore, developing treatment strategies that can provide clinically meaningful improvement within days rather than weeks is imperative (3). Treatment-resistant depression (TRD) is a clinically significant subtype of major depressive disorder, typically defined as failure to respond to at least two adequate antidepressant trials of different classes (3, 4). Compared with non–treatment-resistant depression, TRD has been associated with distinct neurobiological features, including dysregulation of glutamatergic neurotransmission, altered functional connectivity within fronto-limbic networks, and impaired neuroplasticity (5). These differences may partly explain the limited efficacy of conventional monoaminergic antidepressants and have led to the development of alternative therapeutic approaches, including ketamine/esketamine and neuromodulation techniques such as transcranial magnetic stimulation (TMS).

Intranasal esketamine is a fast-acting treatment option for patients with major depressive disorder and suicidal ideation, including those with inadequate responses to conventional antidepressant therapy (6, 7). Additionally, intermittent theta-burst stimulation (iTBS) is an efficient form of transcranial magnetic stimulation (TMS) with a growing body of evidence supporting its antidepressant effects (8), and accelerated iTBS schedules may further shorten the time to response (9). The combination of TMS and ketamine/esketamine treatment has attracted increasing clinical interest for treatment-resistant depression; however, published evidence on this treatment is limited and heterogeneous. For example, Caussat et al. conducted an open-label retrospective cohort study to compare left dorsolateral prefrontal cortex (DLPFC) repeated TMS (rTMS) alone, nasal ketamine alone, and combined rTMS and nasal ketamine treatment in 159 patients with major depressive disorder; they reported greater Patient Health Questionnaire-9 (PHQ-9) score reductions, greater symptom improvement, and higher response rates in the combined-treatment group than in either monotherapy group (10). Additionally, Moleón-Ruiz et al. described a woman in her 60s with electroconvulsive-therapy-resistant depression and suicidal ideation who received combined bilateral theta-burst stimulation (TBS; right continuous TBS [cTBS] plus left iTBS) and intranasal esketamine over 4 weeks; the patient experienced marked improvements in depressive symptoms and suicidality (11). Collectively, these reports demonstrate the feasibility and potential clinical utility of TMS–ketamine/esketamine coadministration.

Despite the preceding findings, critical gaps remain in the literature on combined TMS–ketamine/esketamine treatment. For example, prior studies exhibit substantial heterogeneity in stimulation targets, theta-burst protocols, ketamine/esketamine dosing schedules, and overall treatment duration. Notably, studies on short-duration accelerated inpatient protocols for patients at acutely high risk who require rapid symptom relief are limited, as are practical safety management guidelines for combined treatment.

To address the aforementioned gaps, we present the case of a 29-year-old woman with severe major depressive disorder, high-risk suicidal ideation, and inadequate responses to several pharmacotherapies and a standard course of rTMS; she was treated during hospitalization with a single session of intranasal esketamine combined with an accelerated iTBS protocol (10 sessions over 4 treatment days). Additionally, we report peridose blood pressure management and short-term clinical outcomes, with an exploratory adjunctive device-derived stress index (the Stress Electroencephalogram Assessment [SEA] system) included as a secondary descriptive measure.

## Case description

A 29-year-old single woman was admitted from the outpatient clinic for worsening depressive symptoms, suicidal ideation, insomnia, loss of interest, and feelings of hopelessness. According to her medical record and patient report, her first depressive episode occurred at the age of 21 years, when she was a college senior. She described having depressed mood and suicidal ideation (including thoughts of jumping from a building) associated with sustained workplace-like harassment from a professor (e.g., repeated late-night telephone calls and public verbal intimidation). She was diagnosed with major depressive disorder and treated with escitalopram (10 mg/d titrated to 20 mg/d over 1 month) and psychotherapy, and she experienced gradual symptomatic improvement that enabled her to graduate on time.

In 2024, she developed recurrent and severe symptoms characterized by marked depressed mood, insomnia, helpless, worthless, loss of interest, and suicidal ideation associated with occupational stress (difficulty obtaining leave and perceived punitive supervision); these were accompanied by prominent anticipatory anxiety regarding future job placement. She reported a near-hanging attempt in a school restroom in March 2024 and later described travel to a coastal site with intent to die by suicide. In December 2024, she endorsed renewed suicidal ideation with high-risk behavior (e.g., intentionally accelerating a scooter to approximately 90 km/h near a large truck), which she interrupted due to fear of surviving with severe disabilities. She presented again for psychiatric care and met *Diagnostic and Statistical Manual, Fifth Edition* criteria for major depressive disorder. The patient had no known family history of major depressive disorder or bipolar disorder.

The patient underwent several antidepressant trials at therapeutically accepted doses. According to outpatient medical records, venlafaxine 150 mg/day (75 mg twice daily) had been prescribed continuously from June 30, 2025, until the current hospitalization without satisfactory clinical improvement. The patient also reported previous treatment with escitalopram (20 mg/day), duloxetine (60 mg/day), and augmentation therapy with aripiprazole (5 mg/day) combined with duloxetine (60 mg/day), each administered for more than six weeks at therapeutic doses prior to the current hospitalization. Although complete prescribing records for these earlier treatment trials were unavailable because some treatments were received at outside psychiatric clinics, the patient consistently reported adherence to treatment and inadequate clinical response despite these interventions. Despite these pharmacological interventions, together with structured psychotherapy (including cognitive behavioral therapy) and a subsequent course of more than 30 sessions of repetitive transcranial magnetic stimulation (rTMS), she continued to experience clinically significant depressive symptoms and suicidal ideation. Therefore, she fulfilled the commonly accepted operational definition of treatment-resistant depression, namely inadequate response to at least two antidepressant trials of different pharmacological classes administered at adequate therapeutic doses and durations.

On January 26, 2026, the patient was hospitalized for persistent severe depressive symptoms and suicidal ideation. Baseline symptom severity measures included a PHQ-9 score of 15 (12), a Hamilton Depression Rating Scale score of 27 (13), and a Beck Depression Inventory score of 41 (14).

An exploratory device-derived stress index was obtained using the Stress EEG Assessment (SEA) system (HippoScreen Neurotech) (15), an AI-powered EEG-based assessment tool designed to aid in the evaluation of stress-related brain activity (16, 17). The SEA system has received regulatory approval from the Taiwan Food and Drug Administration (TFDA) as a software as a medical device (SaMD) for assisting in the assessment of suspected depression. Although EEG-based and machine learning–assisted approaches have been increasingly explored in psychiatric research, such tools are not yet standardized for clinical outcome assessment. In this report, the SEA index was included as an adjunctive exploratory measure and was not used as a primary efficacy endpoint. The patient’s initial SEA score was 7.

Esketamine administration was scheduled after admission due to regulatory requirements for controlled substances and the need for prior authorization and monitoring. Given the patient’s limited availability (approximately 7 days), iTBS was initiated prior to esketamine to avoid delay in treatment. Esketamine nasal spray was administered on January 29, 2026 (total dose, 84 mg). The patient had a history of mild hypertension and was receiving amlodipine besylate 5 mg daily. Before esketamine administration, blood pressure was 146/102 mmHg. Following a single oral dose of captopril, blood pressure decreased to 137/101 mmHg. Because of concern regarding potential hypotension with additional antihypertensive treatment, a second dose of captopril was not administered. After discussion of potential risks and benefits, the patient preferred to proceed with esketamine treatment rather than delay administration. Given the severity of depressive symptoms and suicidal ideation, treatment was initiated with close monitoring. Forty minutes after esketamine administration, blood pressure was 141/93 mmHg. No headache, chest pain, neurological symptoms, or other serious adverse events were observed. She reported dissociation that resolved within approximately 2 hours without additional intervention.

Accelerated iTBS was delivered using a MagVenture stimulator with a figure-of-eight coil, with the left DLPFC localized using the Beam F3 method (18, 19). Treatment was scheduled as follows: January 27 (one session), January 28 (three sessions), January 30 (three sessions), and February 2 (three sessions). Each iTBS session comprised 20 trains of 10 bursts (3 pulses at 50 Hz repeated at 5 Hz) at 80% of resting motor threshold, with 8-second intertrain intervals, totaling 600 pulses over 200 seconds per run. After a 50-minute intersession interval, a second run was delivered to the left DLPFC. With the exception of January 27, all daily treatments consisted of three sessions (total, 1800 pulses/d). The full course comprised 6000 pulses.

**Figure 1:**
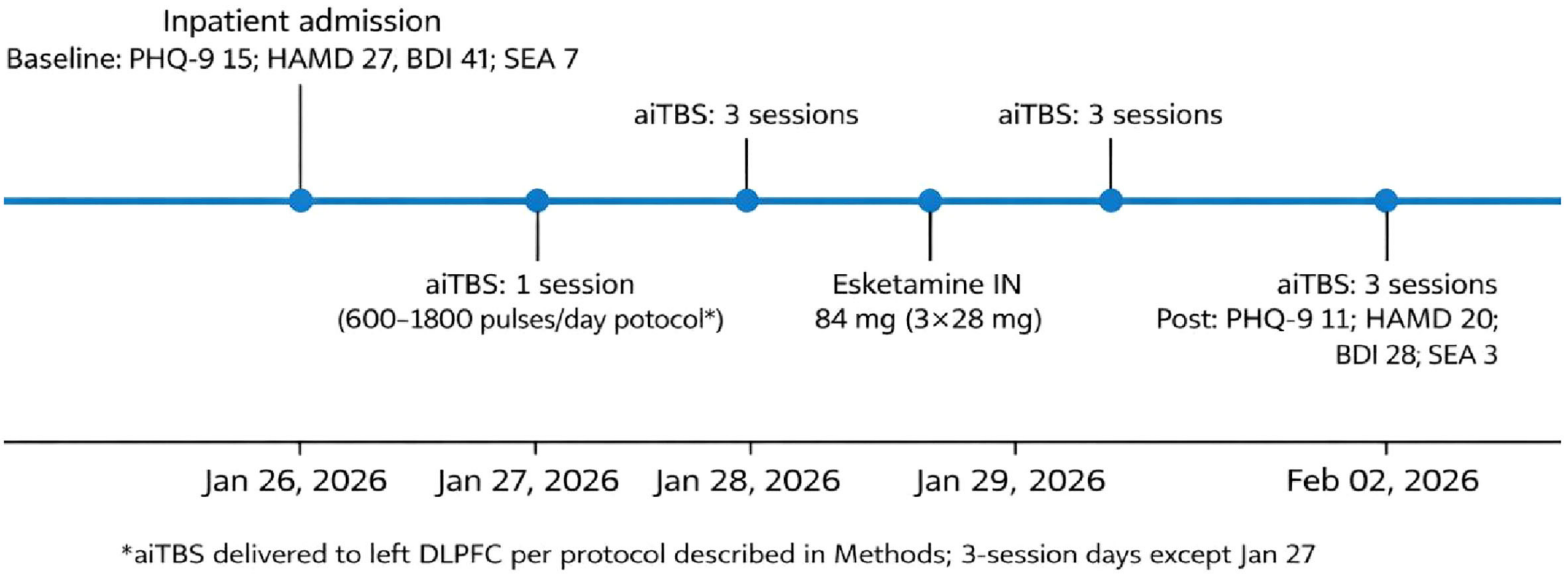
Esketamine + accelerated iTBS timeline.

At reassessment on February 2, 2026, the patient’s SEA index decreased from 7 to 3 (**Supplementary Figure 1**), her PHQ-9 score decreased to 11, her Hamilton Depression Rating Scale score decreased to 20, and her Beck Depression Inventory score decreased to 28. Although depressive symptoms improved, the reduction in scores did not reach the conventional response threshold (≥50% reduction), indicating partial improvement rather than response or remission. The patient’s SEA index decreased, consistent with improvements in depressive symptom ratings. But this index was not used as a primary outcome measure. At a 2-week follow-up, the patient remained clinically stable, with a PHQ-9 score of 8, a Hamilton Depression Rating Scale score of 17, and a Beck Depression Inventory score of 23 (**Figure 1**, **Table 1**).

**Table 1:** Clinical outcomes during treatment.

Symptoms	Before esketamine + accelerated iTBS treatment	After esketamine + accelerated iTBS treatment	Two-week follow-up
PHQ-9	15	11	8
HAMD	27	20	17
BDI	41	28	23

PHQ-9, Patient Health Questionnaire-9. BDI, Beck Depression Inventory. HAMD, Hamilton Depression Rating Scale.

## Discussion

This paper describes the case of a 29-year-old woman with severe major depressive disorder, high-risk suicidal ideation, inadequate responses to several antidepressant trials, and only partial improvements after a standard rTMS course. During inpatient admission, combined intranasal esketamine and an accelerated iTBS protocol targeting the left DLPFC was temporally associated with improvement in depressive symptoms and short-term clinical stability. The magnitude of symptom reduction did not meet the conventional response criterion (≥50% reduction from baseline), and therefore the outcome is best characterized as partial response rather than full response or remission. The classification of TRD in this case was supported by failure to achieve adequate clinical improvement despite multiple antidepressant trials of different pharmacological classes administered at therapeutic doses and durations, augmentation treatment, structured psychotherapy, and a subsequent course of rTMS.

Although the combination of TMS and ketamine/esketamine for treatment-resistant depression (TRD) has been previously reported (20), the present case is not intended to demonstrate novelty at the level of treatment concept. Instead, its primary contribution lies in the extreme simplification of treatment intensity and duration under real-world clinical constraints. In contrast to prior reports that typically involve repeated intranasal esketamine administration (e.g., twice weekly over 4 weeks) combined with multi-week TMS protocols (19), our patient received a single dose of intranasal esketamine and a brief accelerated iTBS course completed within one week. For example, Moleón-Ruiz et al. described a case requiring eight esketamine administrations over four weeks combined with bilateral theta burst stimulation, representing a substantially longer and more intensive treatment schedule (11).

Importantly, the abbreviated regimen in the present case was not designed as an experimental optimization strategy but rather emerged from pragmatic real-world constraints, including limited patient availability (only one week of leave) and insurance coverage restricted to a single esketamine administration. Despite these limitations, the patient demonstrated partial clinical improvement, suggesting that the minimal effective intensity and duration of combined esketamine–iTBS treatment remain uncertain and warrant systematic investigation. While no causal inference can be drawn from a single case, this observation raises the possibility that dose and schedule optimization—particularly toward shorter and less intensive regimens—may be feasible and warrants further investigation.

This perspective is consistent with broader patterns in the evolution of medical treatments. The antidepressant effect of ketamine itself was originally identified when the dose was reduced from anesthetic to sub-anesthetic levels (21). Similarly, TMS protocols have evolved from conventional 6-week courses to accelerated paradigms such as the Stanford Accelerated Intelligent Neuromodulation Therapy (SAINT), which delivers multiple daily sessions over 5 days with promising outcomes (22, 23). More generally, the concept of “shorter is better” has been demonstrated in other fields of medicine, such as antibiotic therapy, where shorter treatment durations have been shown to maintain efficacy while reducing adverse effects and resistance (24). .

Several studies have reported TMS–ketamine/esketamine coadministration (**Table 2**). For example, Best et al. reported a retrospective case series of 28 patients receiving coincident intravenous (IV) ketamine plus TMS in a private practice setting. Their study used a prolonged and highly individualized protocol (typically 10–30 sessions) with medial prefrontal/Fz-targeted low-frequency stimulation and ketamine infusion titrated to induce a mildly cataleptic state, and outcomes were primarily assessed through clinician-rated clinical global impression scores with long-term follow-up (25). Similarly, Caussat et al. provided retrospective real-world evidence in a larger cohort (*N* = 159) and demonstrated superior PHQ-9 scores and response rates with concurrent left DLPFC rTMS plus nasal ketamine versus either treatment alone; nevertheless, their report was an abstract-level cohort analysis with limited procedural information and details of safety monitoring (10). By contrast, Shanok et al. evaluated a nonconcurrent augmentation model in which 36 sessions of deep TMS (H1 + H7 protocols) were combined with 6 IV ketamine infusions over 9 weeks; they noted no significant added antidepressant benefit over deep TMS alone, despite robust improvements in both groups (26). Our case study differs from these three studies in several respects. First, it focused on an acutely high-risk inpatient presentation with suicidal ideation. Second, it used a brief accelerated iTBS protocol (10 sessions over 4 treatment days) targeted to the left DLPFC (beam F3) combined with a single intranasal esketamine session. These differences reveal that protocol timing, stimulation targets, ketamine formulation/route, treatment intensity, and clinical setting can substantially influence feasibility and observed benefits in combined TMS–ketamine/esketamine treatment strategies for major depressive disorder.

**Table 2:** Summary of studies on rTMS and esketamine/ketamine for major depressive disorder.

Study	Sample size	Depression measure tool	Mean age	TMS protocol	Ketamine protocol	Study outcome
Caussat et al. (2023) (10)	*N* = 159 (rTMS only: 86; nKet only: 30; Combined: 43)	PHQ-9	Not reported	Target: Left DLPFC Timing: Concurrent with Ketamine	Type: Nasal Ketamine (nKet) Timing: Concurrent (administered during TMS)	Combined therapy was superior. Coadministration resulted in significantly greater symptom reduction (61.32%) relative to rTMS (50.22%) or nKet (38.38%) alone. Response rates were also significantly higher in the combined group
Best et al. (2019) (25)	*N* = 28 (All patients received combined therapy)	CGI-S, CGI-I	41.0 ± 15.6 years	Freq: 1 Hz (Low Frequency) Intensity: 130% MT Target: Medial PFC/ACC (Fz)Duration: 30 minutes (continuous)	Type: IV infusionDose: Titrated to catalepsy (0.4–2.3 mg/kg) Timing: Concurrent (Start 5 minutes after TMS began)	Significant, sustained remission. CGI-S scores decreased from 6.1 (pre) to 1.7 (post) and remained low (1.4) at a 2-year follow-up. Ketamine sedation allowed patients to tolerate higher-than-normal TMS intensities.
Shanok et al. (2024) (26)	*N* = 235 (TMS only: 169; TMS + Ketamine: 66)	PHQ-9	Overall: 52.54 TMS + Ket: 51.84 TMS only: 52.78	Device: Deep TMS (H1 + H7 Coils) Freq: 18 Hz (High Frequency) Target: Left DLPFC and Medial PFC/ACC Sessions: 36 (over 9 weeks)	Type: IV (0.5 mg/kg) + Subcutaneous (0.1 mg/kg) Sessions: 6 infusions Timing: During the 9-week TMS course (not strictly concurrent)	No added benefit from ketamine. Both groups exhibited high response rates (77%–80%), and adding six ketamine sessions to the deep TMS protocol did not yield significant differences in response or remission rates compared with deep TMS alone

CGI-S, Clinical Global Impression–Severity; PHQ-9, Patient Health Questionnaire-9; HDRS-17, 17-item Hamilton Depression Rating Scale; DLPFC, dorsolateral prefrontal cortex; ACC, anterior cingulate cortex; MT, motor threshold; iTBS, intermittent theta-burst stimulation.

The sequence of iTBS administered before and after intranasal esketamine in this case was primarily determined by pragmatic clinical constraints rather than a predefined experimental design. Esketamine is a controlled medication in our setting and requires prior authorization and coordinated safety monitoring, precluding administration on the day of admission. In addition, the patient’s limited availability (approximately 7 days) necessitated prompt initiation of treatment, leading to the start of iTBS prior to esketamine and continuation of iTBS thereafter.

From a mechanistic perspective, both iTBS and esketamine are thought to modulate glutamatergic neurotransmission and NMDA receptor–related pathways (27, 28), raising the possibility of temporally dependent or bidirectional synergistic effects. However, the present case was not designed to evaluate sequencing effects, and therefore any inference regarding synergy should be considered exploratory. Future studies are warranted to systematically examine the optimal temporal sequencing of neuromodulation and glutamatergic interventions.

The clinical rationale for this combined approach was the need for timely symptom relief and the imminent risk to patient safety, as well as the desire to provide a structured course of neuromodulation during hospitalization. Intranasal esketamine provides a fast-acting antidepressant option (28), and iTBS is an efficient form of TMS; accelerated schedules may also shorten time to response (9, 29). One plausible explanation for the effectiveness of the combined TMS–ketamine/esketamine treatment approach is that rapid glutamatergic modulation and downstream synaptic plasticity associated with esketamine could create a favorable neurobiological period during which repeated iTBS sessions support network-level changes in frontolimbic circuits implicated in mood regulation (30). However, such mechanistic synergy remains hypothetical and warrants prospective evaluation.

The safety and tolerability of the combined treatment approach were clinically salient. Acute adverse effects of esketamine (e.g., dizziness, dissociation, and hypertension) were transient and resolved within approximately 2 hours. No unexpected complications were observed during the accelerated iTBS schedule. Notably, the patient expressed a clear preference to proceed without delaying esketamine after discussion of the potential risks and benefits, and she reported improvements in both depressive symptoms and suicidal ideation after treatment. Our clinical practice follows the recommendations outlined in the SPRAVATO^®^ prescribing information, which advises individualized risk-benefit assessment and blood pressure monitoring rather than an absolute blood pressure cutoff (31, 32). This case highlights the importance of individualized cardiovascular risk assessment and shared decision-making when administering esketamine in patients with mildly elevated baseline blood pressure.

This case report has several limitations. First, causality cannot be inferred from a single case, and symptom improvement may have been influenced by nonspecific factors such as inpatient containment, expectancy effects, or the natural course of illness. Second, the follow-up period was limited to 2 weeks; therefore, conclusions regarding treatment durability, relapse risk, or the need for maintenance strategies could not be drawn. Third, the SEA system represents an emerging class of AI-assisted EEG-based tools; however, such measures remain exploratory and are not yet validated as standardized biomarkers for depression severity or treatment response. Fourth, the heterogeneity of psychotherapy modalities and the lack of standardized documentation limit interpretation of its contribution to clinical outcomes in this case. Although detailed prescribing records from outside clinics were unavailable, the prolonged venlafaxine treatment documented in our medical records and the patient’s consistent report of prior antidepressant exposure support the clinical classification of TRD.

In summary, for a patient with major depressive disorder who is at acutely high risk of suicidal ideation and has inadequate responses to prior treatments, a combination of intranasal esketamine and accelerated iTBS was feasible in an inpatient setting with blood pressure monitoring; the combined treatment approach was associated with rapid symptom improvement and short-term stability. Controlled studies are required to determine the therapeutic efficacy, optimal sequencing, safety monitoring parameters (including management of peridose hypertension), and longer-term outcomes of this combined approach in similar high-risk populations.

## Conclusion

In this inpatient case of severe major depressive disorder with high-risk suicidal ideation, combined intranasal esketamine and accelerated iTBS was feasible and well tolerated. The patient demonstrated partial clinical improvement, with reductions in depressive symptoms and suicidal ideation, and maintained short-term stability at 2-week follow-up. Given the single-case design, these findings should be interpreted cautiously. Further controlled studies are needed to clarify efficacy, optimal sequencing, and long-term outcomes.

## Data Availability

The original contributions presented in the study are included in the article/**Supplementary Material**. Further inquiries can be directed to the corresponding author.

## Supplementary material

Supplementary Figure 1 **(A)** Pretreatment and **(B)** posttreatment stress electroencephalogram assessment system outputs. Representative pretreatment (score = 7) and posttreatment (score = 3) outputs from the Stress Electroencephalogram Assessment (SEA) system (HippoScreen Neurotech). The SEA is an artificial-intelligence-powered measure with Taiwan Food and Drug Administration regulatory approval as software as a medical device, and is used to assess suspected depression. In the present case, the patient’s SEA score decreased after treatment, consistent with improvements in other depressive symptom ratings. The SEA output was included as an adjunctive exploratory measure and was not used as a primary outcome measure.
