# Supplementary material for: Case Report: Intranasal esketamine and accelerated intermittent theta-burst stimulation for severe treatment-resistant depression with suicidal ideation
Source: Front Psychiatry. 2026 Jul 1;17:1837402. doi: 10.3389/fpsyt.2026.1837402 (PMC13373716; doi:10.3389/fpsyt.2026.1837402)
Supplement: Supplementary file 1 [file Table1.docx]

Supplement Table 1. Timeline of intranasal esketamine and accelerated iTBS treatment.

The patient received 10 iTBS sessions over 4 treatment days (January 27, 28, 30, and February 2, 2026), with a total of 6000 pulses delivered to the left DLPFC. On days with multiple sessions, iTBS treatments were separated by approximately 50-minute intersession intervals. A single dose of intranasal esketamine (84 mg) was administered on January 29, 2026.

| **Date** | **Day** | **Esketamine** | **iTBS Sessions** | **Total Pulses** | **Notes** |
| --- | --- | --- | --- | --- | --- |
| Jan 27, 2026 | Day 1 | — | 1 session | 600 | Initiation of iTBS |
| Jan 28, 2026 | Day 2 | — | 3 sessions | 1800 | Sessions separated by 50-minute intervals |
| Jan 29, 2026 | Day 3 | 84 mg | — | — | Esketamine administration |
| Jan 30, 2026 | Day 4 | — | 3 sessions | 1800 | Sessions separated by 50-minute intervals |
| Jan 31 – Feb 1 | — | — | — | — | No treatment |
| Feb 2, 2026 | Day 5 | — | 3 sessions | 1800 | Sessions separated by 50-minute intervals |
| **Total** | — | **84 mg** | **10 sessions** | **6000 pulses** | — |
