# Supplementary material for: Case Report: Intranasal esketamine and accelerated intermittent theta-burst stimulation for severe treatment-resistant depression with suicidal ideation
Source: Front Psychiatry. 2026 Jul 1;17:1837402. doi: 10.3389/fpsyt.2026.1837402 (PMC13373716; doi:10.3389/fpsyt.2026.1837402)

Supplementary Image 1. (a) Pretreatment and (b) posttreatment stress electroencephalogram assessment system outputs

Representative pretreatment (score = 7) and posttreatment (score = 3) outputs from the Stress Electroencephalogram Assessment (SEA) system (HippoScreen Neurotech). The SEA is an artificial-intelligence-powered measure with Taiwan Food and Drug Administration regulatory approval as software as a medical device, and is used to assess suspected depression. In the present case, the patient's SEA score decreased after treatment, consistent with improvements in other depressive symptom ratings. The SEA output was included as an adjunctive exploratory measure and was not used as a primary outcome measure.

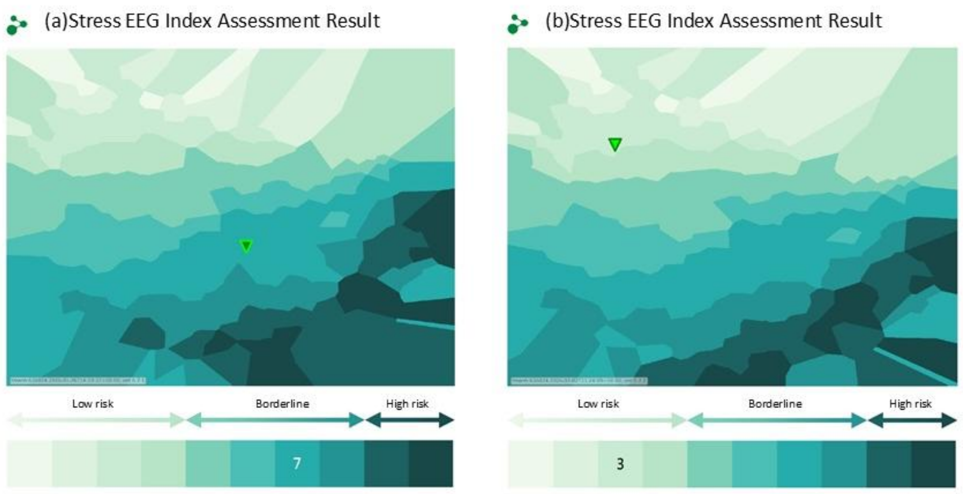

Supplement: Supplementary Figure 1 — (A) Pretreatment and (B) posttreatment stress electroencephalogram assessment system outputs. Representative pretreatment (score = 7) and posttreatment (score = 3) outputs from the Stress Electroencephalogram Assessment (SEA) system (HippoScreen Neurotech). The SEA is an artificial-intelligence-powered measure with Taiwan Food and Drug Administration regulatory approval as software as a medical device, and is used to assess suspected depression. In the present case, the patient’s SEA score decreased after treatment, consistent with improvements in other depressive symptom ratings. The SEA output was included as an adjunctive exploratory measure and was not used as a primary outcome measure. [file SupplementaryFile1.pdf]
